# The M1/M2 spectrum and plasticity of malignant pleural effusion-macrophage in advanced lung cancer

**DOI:** 10.1007/s00262-020-02781-8

**Published:** 2020-11-11

**Authors:** Ming-Fang Wu, Chih-An Lin, Tzu-Hang Yuan, Hsiang-Yuan Yeh, Sheng-Fang Su, Chin-Lin Guo, Gee-Chen Chang, Ker-Chau Li, Chao-Chi Ho, Huei-Wen Chen

**Affiliations:** 1 Graduate Institute of Toxicology, College of Medicine, National Taiwan University, No.1, Section 4, Ren-Ai Rd, Taipei, 100 Taiwan; 2 Institute of Statistical Sciences, Academia Sinica, Taipei, Taiwan; 3 Department of Internal Medicine, National Taiwan University Hospital and National Taiwan University College of Medicine, No. 7 Chung-Shan South Road, Taipei, 100 Taiwan; 4 Genome and Systems Biology Degree Program, National Taiwan University and Academia Sinica, Taipei, 100 Taiwan; 5 School of Big Data Management, Soochow University, Taipei, Taiwan; 6 Graduate Institute of Oncology, National Taiwan University College of Medicine, Taipei, Taiwan; 7 Institute of Physics, Academia Sinica, Taipei, Taiwan; 8 Division of Chest Medicine, Department of Internal Medicine, Taichung Veterans General Hospital, Taichung, Taiwan; 9 Faculty of Medicine, School of Medicine, National Yang-Ming University, Taipei, Taiwan; 10 Institute of Biomedical Sciences, National Chung Hsing University, Taichung, Taiwan; 11 Division of Pulmonary Medicine, Department of Internal Medicine, Chung Shan Medical University Hospital, Taichung, Taiwan; 12 Department of Statistics, University of California, Los Angeles, Los Angeles, CA 90095 USA

**Keywords:** Malignant pleural effusion, Macrophages, Lung cancer

## Abstract

**Background:**

Malignant pleural effusion (MPE)-macrophage (Mφ) of lung cancer patients within unique M1/M2 spectrum showed plasticity in M1–M2 transition. The M1/M2 features of MPE-Mφ and their significance to patient outcomes need to be clarified; furthermore, whether M1-repolarization could benefit treatment remains unclear.

**Methods:**

Total 147 stage-IV lung adenocarcinoma patients undergoing MPE drainage were enrolled for profiling and validation of their M1/M2 spectrum. In addition, the MPE-Mφ signature on overall patient survival was analyzed. The impact of the M1-polarization strategy of patient-derived MPE-Mφ on anti-cancer activity was examined.

**Results:**

We found that MPE-Mφ expressed both traditional M1 (HLA-DRA) and M2 (CD163) markers and showed a wide range of M1/M2 spectrum. Most of the MPE-Mφ displayed diverse PD-L1 expression patterns, while the low PD-L1 expression group was correlated with higher levels of IL-10. Among these markers, we identified a novel two-gene MPE-Mφ signature, IL-1β and TGF-β1, representing the M1/M2 tendency, which showed a strong predictive power in patient outcomes in our MPE-Mφ patient cohort (*N* = 60, *p* = 0.013) and The Cancer Genome Atlas Lung Adenocarcinoma dataset (*N* = 478, *p* < 0.0001). Significantly, β-glucan worked synergistically with IFN-γ to reverse the risk signature by repolarizing the MPE-Mφ toward the M1 pattern, enhancing anti-cancer activity.

**Conclusions:**

We identified MPE-Mφ on the M1/M2 spectrum and plasticity and described a two-gene M1/M2 signature that could predict the outcome of late-stage lung cancer patients. In addition, we found that “re-education” of these MPE-Mφ toward anti-cancer M1 macrophages using clinically applicable strategies may overcome tumor immune escape and benefit anti-cancer therapies.

**Electronic supplementary material:**

The online version of this article (10.1007/s00262-020-02781-8) contains supplementary material, which is available to authorized users.

## Introduction

Pleural effusion (PE) is a common clinical problem caused by malignant cancers [1, 2]. Malignant pleural effusion (MPE), which is correlated with cancer-induced morbidity and mortality, occurs in advanced lung cancer patients and results in reduced life quality for those patients [3]. The cellular microenvironment of MPE is essential for malignant cancer progression, metastasis, and immune dysfunction. However, the underlying mechanism has not been adequately studied [4–6]. Tumor-associated macrophages (TAMs), which could be major population in MPE, often increase chemo-resistance and regulate immunity by reducing T cell activation, potentially through the interaction of programmed cell death protein 1 (PD-1) and programmed death-ligand 1 (PD-L1) or through the release of immunosuppressive molecules, such as transforming growth factor-β (TGF-β) and interleukin (IL)-10 [5, 7, 8].

Macrophages (Mφ) are divided into classical M1 or alternative M2 types based on differential activation signals in tumor microenvironments. M1 macrophages with elevated HLA-DR produce proinflammatory cytokines, such as IL-1β and tumor necrosis factor (TNF), to kill tumors, while M2 macrophages with increased scavenger receptor (CD163) and mannose receptor (MRC1, CD206) to release immunosuppressive molecules, such as IL-10 and TGF-β, to promote tumor growth [9–11]. Immune escape in the tumor microenvironment has been reported to repolarize macrophages from M1 during tumor initiation to M2 macrophages in the late stage of tumor progression and metastasis [12]. M1/M2 repolarization in non-small cell lung carcinoma (NSCLC) patients has been suggested as a potential prognostic factor for survival [13, 14].

Previous studies indicated that CD14^+^ CD163^+^ macrophages could be used as diagnostic markers for MPE [15]. In addition, MPE-derived CD163^+^ TAMs were associated with progression-free survival (PFS) in lung cancer [16]. These findings raised the possibility that CD14^+^CD163^+^ M2 TAMs could be the dominant macrophage population in MPE and suggested that targeting CD14^+^ CD163^+^ M2 TAMs may result in an effective therapy for tumor progression. However, a comprehensive analysis of the M1/M2 patterns in MPE remains to be determined and whether MPE-Mφ could be repolarized into anti-cancer M1 macrophages needs to be validated.

β-glucans, a group of polysaccharides majorly exist in mushrooms and seaweed, have many applications in drugs and healthcare products; recently, as a potential immune adjuvant [17] via their ability to trigger proinflammatory cytokine release, reactive oxygen species (ROS) production, and phagocytosis through the Dectin-1/Syk signaling pathway in macrophages [18]. β-glucan could repolarize mouse tissue macrophages and bone marrow-derived M2 macrophages into M1-like macrophages [19]. Furthermore, interferon-gamma (IFN-γ)-stimulated macrophages, which were referred to as M1 macrophages or M (IFN-γ) cells [20], could decrease mouse lung cancer growth by decreasing the M2/M1 ratio in tumor microenvironments [21]. However, it is unknown whether β-glucan or IFN-γ have the potential to repolarize patient-derived MPE-Mφ from M2 to M1.

In this study, we sought to clarify the unique signature of MPE-Mφ in advanced lung adenocarcinoma patients and tried to target these Mφ for prognostic prediction or therapeutic intervention. We found that MPE-Mφ from different patients were heterogeneous with a wide spectrum of M1/M2 marker expression patterns and less correlation with the level of the immune checkpoint, PD-L1. Significantly, we identified a novel two-gene MPE-Mφ signature (IL-1β and TGF-β1) representing the M1/M2 tendency of the MPE-Mφ in each individual case, which showed a strong predictive power in patient overall survival (OS) in both our National Taiwan University Hospital (NTUH) cohort (*N* = 60) and the Cancer Genome Atlas Lung Adenocarcinoma (TCGA-LUAD) dataset (*N* = 478) [22]. Additionally, we aimed to demonstrate that the plastic MPE-Mφ could be “educated” to benefit critical anti-cancer therapies. Importantly, our results revealed a surprising potential of IFN-γ and β-glucan in the conversion of MPE-Mφ from M2 (TAM) tendency toward anti-tumor phenotypes (M1), which could be helpful for improving anti-tumor immunity in advanced lung cancer patients.

## Materials and methods

### Antibodies, reagents, and cell lines

Anti-CD14 antibody was purchased from BD, while anti-CD68, anti-CD163, anti-HLA-DR antibodies and isotype controls for flow cytometry were purchased from Biolegend. A LIVE/DEAD™ Fixable Dead Cell Stain Kit was purchased from Thermo Fisher. Recombinant IFN-γ, TNF-α, IL-10, IL-13 and TGF-β1 were purchased from Peprotech. Recombinant granulocyte–macrophage colony-stimulating factor (GM-CSF), macrophage colony-stimulating factor (M-CSF), IL-4, and neutralizing IL-10 antibody were from R&D. Neutralizing IFN-γ and TNF-α antibodies were from Biolegend. Mouse IgG1 (Biolegend) and mouse IgG2b (R&D) were used as isotype-matched antibody controls for neutralization studies. β-Glucan was purchased from Sigma. All other materials, unless specifically mentioned, were purchased from Sigma-Aldrich. The CLS1 cell line was originally established from a lung cancer patient with NSCLC and had characteristics of cancer stem cells in our previous study [23] and has been found to have KRAS (Q61H) and PIK3CA (E545K) mutations. A549 was obtained from the National Cancer Institute (National Institutes of Health, Bethesda, MD, USA). These cells were cultured in RPMI medium supplemented with 10% fetal bovine serum (FBS) at 37 °C in 5% CO_2_.

### Collection of malignant pleural effusions (MPEs)

MPEs were collected from 147 patients with stage IV lung adenocarcinoma at National Taiwan University Hospital (NTUH) from 2008 to 2020, based on the institutional review board (IRB) protocol of the NTUH Research Ethics Committee (approval number: 200709004R). Written informed consent was collected from all patients. Among these 147 samples, 60 MPE samples from 2008 to 2011 were characterized for M1 and M2 and macrophage gene expression; 87 MPE samples from 2016 to 2020 were used for the validation of M1 and M2 expression (flow cytometry) and repolarization studies.

### Isolation of macrophages from malignant pleural effusion Mφ (MPE-Mφ)

CD14^+^ macrophages in MPEs were isolated by high-gradient magnetic separation technique with a MACS system using anti-CD14 microbeads (Miltenyi Biotec)**.** These were considered day 0 (D0) MPE-Mφ.

### Flow cytometric analysis

MPE-Mφ were incubated with fluorochrome-labeled antigen-specific mAbs or isotype controls in phosphate buffered saline (PBS) at 4 °C for 30 min. Cells were then washed twice with PBS, and then analyzed by flow cytometry on an LSRFortessa (Becton Dickinson). Live or dead cells were distinguished by staining with a live/dead fixable dye.

### Gene expression analysis and heatmap

Expression of MPE-Mφ genes was validated by real-time PCR, calculated as 2^−∆Ct^ (∆Ct = Ct of target gene − Ct of internal control) and normalized by log2 transformation (Supplementary Table 1 for primer sequences). The TCGA database was selected and normalized as described previously [22, 24]. Reference M1 and M2 macrophage gene expression data were selected from the NCBI GEO series GSE5099. The heatmap of the gene expression data was generated using the R package “ComplexHeatmap” [25].

### Statistical analysis

All in vitro experiments were performed with the indicated cases as described in the figure legends and the statistical analysis for the data was evaluated with unpaired/paired *t* tests or the Mann–Whitney *U* test (PRISM software package, GraphPad 8.0). Other statistical analyses, including survival presentation and the univariate and multivariate Cox proportional model, are described in “Supplementary Materials”.

## Results

### Heterogeneous expression of M1 and M2 markers by MPE-Mφ from advanced lung cancer patients

**Fig. 1:**
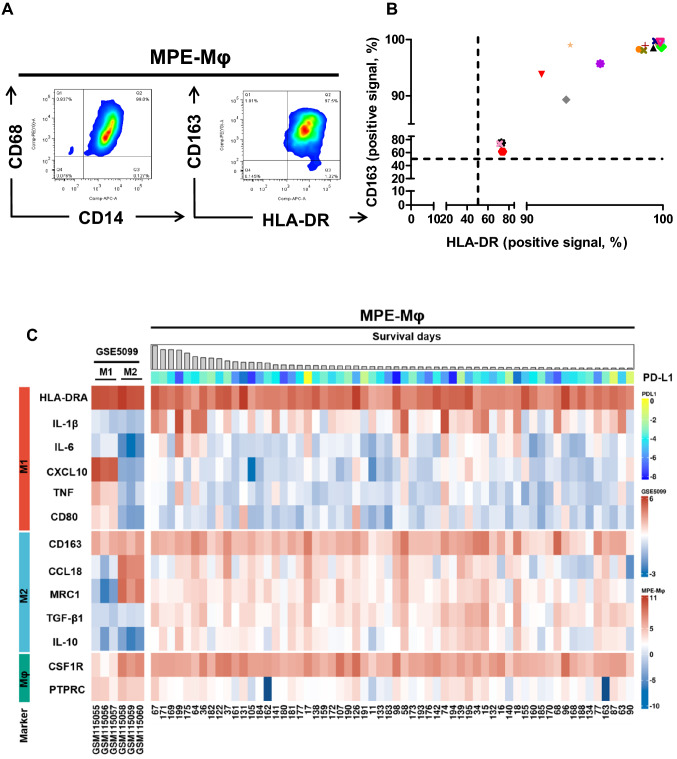

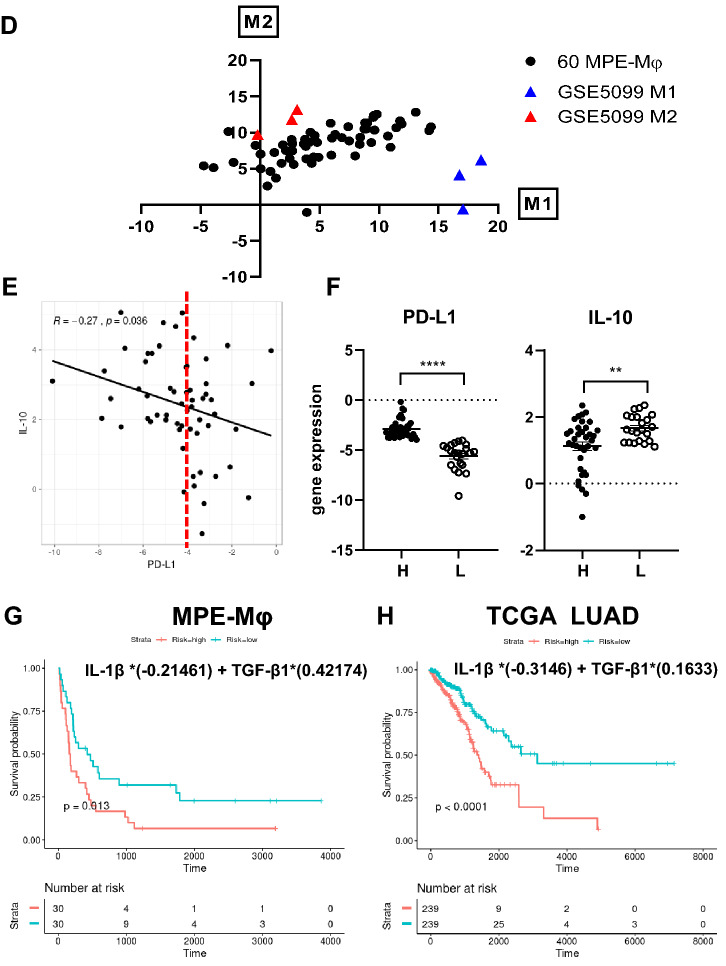
M1 and M2 markers are heterogeneously expressed in malignant pleural effusion-macrophages (MPE-Mφ) of advanced lung cancer patients. **a** MPE-Mφ with the common macrophage marker CD68 were double-positive for the expression of M1 (HLA-DR) and M2 (CD163) markers. MPE-Mφ isolated using anti-CD14 microbeads were characterized by flow cytometry with CD14, CD68, HLA-DR, and CD163 staining. Gating was based on isotype control staining. **b** The coexpression of M1 (HLA-DR) and M2 markers (CD163) by MPE-Mφ from 16 MPE samples was further evaluated by flow cytometry. One point indicates 1 MPE-Mφ case. **c** Heatmap of M1, M2 and common macrophage marker gene expression for 60 MPE-Mφ and for reference M1 and M2 macrophages (NCBI GEO series GSE5099). The arrangement of the 60 MPE-Mφ subjects was based on survival days, and programmed death-ligand 1 (PD-L1) expression levels for patients are also shown under the survival days for the indicated patients. Sample numbers of MPE-Mφ (*N* = 60) or triplicates of reference macrophages are listed in the columns. Gene expression levels are illustrated in a color gradient; red/yellow indicates high expression, while blue indicates low expression, as shown in the right panel. The names of the genes in each cluster can be seen in the left panel. **d** The M1/M2 gene expression distribution for MPE-Mφ and reference M1 and M2 macrophages (GEO database, GSE5099). As described in “Materials and methods”, the sum expression for six M1 genes (*x*-axis, M1) and five M2 genes (*y*-axis, M2) was plotted in the figure. Black closed circles indicate the 60 MPE-Mφ samples, individually; blue closed triangles indicate the triplicate GSE5099 M1 macrophages; red closed triangles indicate the triplicate GSE5099 M2 macrophages. **e** The correlation between IL-10 and PD-L1 was studied by constructing a scatterplot with a linear regression. IL-10 gene expression negatively correlated with PD-L1 was selected from Supplementary Fig. 2 and a scatterplot with PD-L1 was generated using the R package ggpubr [44] and ggplot2 [45]. The correlation (*R*) and *p* value are shown in the figure. One dot indicates an individual subject (*N* = 59, no. 18 listed in **c** was ruled out because of undetectable PD-L1 expression). The red dotted line (PD-L1 = − 4) separates the MPE-Mφ samples into PD-L1 high (*N* = 37) and low populations (*N* = 22). **f** The gene expression of PD-L1 and IL-10 in PD-L1 high and low populations. The PD-L1 gene expression levels of the MPE-Mφ subjects were divided into PD-L1 high and low populations as shown in **e**. H, PD-L1 high population (*N* = 37); L, PD-L1 low population (*N* = 22). Data were expressed as the means ± SEM and compared using an unpaired *t* test; **p < 0.01, ****p < 0.0001. **c**–**f** Expression of MPE-Mφ genes was calculated and normalized as described in “Materials and methods”. **g**–**h** Kaplan–Meier estimates of NSCLC patient survival according to the indicated M1 (IL-1β) and M2 (TGF-β1) gene signature of their MPE-Mφ. **g** Kaplan–Meier curves of the overall survival (OS) for NSCLC according to the indicated M1 (IL-1β) and M2 (TGF-β1) gene signature of their MPE-Mφ. *N* = 60; log-rank test, *p* = 0.013. **h** Kaplan–Meier curves of OS for the Cancer Genome Atlas Lung Adenocarcinoma (TCGA-LUAD) data stratified by M1 (IL-1β) and M2 (TGF-β1) gene expression. *N* = 478; log-rank test, *p* < 0.0001

MPE-Mφ collected from lung cancer patients were identified as CD14^+^ CD68^+^ macrophages (Fig. 1a, left panel). Of interest, we found that all of the isolated MPE-Mφ were double-positive for M1 (HLA-DR) and M2 (CD163) markers (Fig. 1a, right panel) and showed high correlation, but heterogeneity in different patients (Fig. 1b). The M1/M2 tendency might be correlated with the immunity and prognosis of the patients. To further clarify the heterogeneity of the M1/M2 patterns of the MPE-Mφ, we collected MPE-Mφ from advanced lung adenocarcinoma patients (*N* = 60), and the gene expression of six M1 markers (HLA-DRA, IL-1β, IL-6, CXCL10, TNF, and CD80), five M2 markers (CD163, CCL18, MRC1, TGF-β1, and IL-10) and two pan-macrophage markers (CSF1R and PTPRC) were characterized. As shown in Fig. 1c, a heatmap showed heterogeneity in the expression patterns of the M1, M2, and pan-macrophage markers of the 60 MPE-Mφ samples. Most of the MPE-Mφ had relatively higher expression levels of HLA-DRA (M1), CD163 (M2) and the pan-macrophage marker CSF1R; compared to the reference patterns of the classical M1 and M2 macrophages from the GEO database (GSE5099). Comparing to the reference M1 and M2 macrophages (GSE5099), the sums of the expression of the six M1 (*x*-axis) and five M2 genes (*y*-axis) indicated that M1/M2 markers of the MPE-Mφ is continuous across the spectrum of M1 and M2, with a tendency toward the M2 phenotype (Fig. 1d). In addition, we collected CD14^+^ monocytes from peripheral blood mononuclear cells (PBMCs) of lung cancer patients and healthy controls (HCs), and differentiated them into macrophages via incubation with M-CSF. The M1 and M2 phenotypes of the monocyte-derived macrophages (MDMs) were compared with the MPE-Mφ (Supplementary Fig. 1). MPE-Mφ showed relatively higher expression levels of M1 (IL-1β, IL-6, and CXCL10), M2 (CCL18 and IL-10), and pan-macrophage markers (CSF1 and PTPRC) compared to MDMs from lung cancer patients or HCs. Interestingly, comparing to the MDMs from HCs, some cases of lung cancer patients’ MDMs showed MPE-Mφ-like patterns on elevating expression levels of CXCL10, CCL18, and MRC1, may indicate the local and systemic effects of the tumor microenvironment.

Previous study showed that blockage of PD-L1 might promote TAMs into a more proinflammatory state [26]. Here, we aim to investigate the correlation of PD-L1 and M1/M2 polarization. Figure 1c showed that although PDL1 showed relative low expression level among these 60 MPE-Mφ samples. We found that the expression level of 3 genes (IL-10, TNF and CD80) had a significant negative correlation with PD-L1 on the MPE-Mφ (Fig. 1e, Supplementary Figs. 2 and 3). Interestingly, the Fig. 1e scatter plot indicated that a cutoff of -4 for PD-L1 could distinguish two subpopulations of the IL-10 expression. Based on this cutoff strategy, we divided MPE-Mφ into two groups (High, H; Low, L) according to the PD-L1 expression (Fig. 1f, left panel). The right panel of Fig. 1f demonstrates that there was higher IL-10 expression in the PD-L1 low group and lower IL-10 expression in the PD-L1 high group (*p* < 0.01). In contrast, other M1, M2 or pan-macrophage markers among the PD-L1 high and low groups showed no significant difference (Supplementary Fig. 4).

### M1 (IL-1β) and M2 (TGF-β1) two-gene signature relative to OS of NSCLC patients

Whether M1 and M2 markers can be used as prediction markers for clinical outcomes is still controversial. Here, we found that two (IL-1β and TGF-β1) of the 13 genes showed the strongest correlations with patient OS (*p* < 0.01). The Cox coefficients of the IL-1β and TGF-β1 genes among MPE-Mφ and TCGA-LUAD subjects were calculated (Supplementary Table 2). Based on this M1/M2 2-gene signature (IL-1β, M1 marker and TGF-β1, M2 marker), the Cox high-risk population (more like the M2 population) and low-risk population (more like the M1 population) were separated according to the median and calculated the significance between both groups (*p* = 0.013) (Fig. 1g) (hazard ratio (H.R.) for the risk score was 2.022; 95% confidence interval (C.I.) 1.146–3.566, *p* = 0.0151). We also validated this M1/M2 2-gene signature using the TCGA-LUAD dataset [22] (Fig. 1h), and found a significant association with patient outcome, in that those with a high-risk gene signature had shorter OS than those with a low-risk gene signature (*p* < 0.0001) (H.R. for the risk score was 2.26; 95% C.I. 1.543–3.309, *p* = 0.0000279). Furthermore, the univariate and multivariate Cox proportional analyses revealed that the high-risk group was an independent risk for mortality (crude H.R. 2.021 (95% C.I. 1.146–3.566) and adjusted 1.918 (95% C.I. 1.025–3.587)) (Table 1).

**Table 1:** Univariate and multivariate Cox proportional hazard regression for mortality analysis (*N* = 60)

Characteristics	Univariate		Multivariate^c^	
	H.R. (95% C.I.)^a^	*p* value	H.R. (95% C.I.)^a^	*p* value
Risk group^b^				
Low	Reference		Reference	
High	2.021 (1.146–3.566)	0.015	1.918 (1.025–3.587)	0.041
Age, year	1.009 (0.983–1.036)	0.508	1.001 (0.974–1.029)	0.923
Gender, male vs. female	1.079 (0.618–1.883)	0.790	1.142 (0.622–2.097)	0.669
EGFR gene mutation				
None	Reference		Reference	
L858R or Del 19 or G719D	1.397 (0.748–2.610)	0.294	1.147 (0.596–2.208)	0.682
Unknown	0.581 (0.210–1.604)	0.295	0.466 (0.162–1.342)	0.157
Treatment				
None	Reference		Reference	
Targeted therapy	1.653 (0.689–3.966)	0.260	1.957 (0.752–5.095)	0.169
Chemotherapy	0.989 (0.538–1.819)	0.972	0.895 (0.478–1.673)	0.727
Targeted therapy + Chemotherapy	0.222 (0.029–1.677)	0.145	0.242 (0.031–1.908)	0.178

^a^C.I., confidence interval, H.R., hazard ratio

^b^Risk groups were classified according to the two-gene signatures [IL-1β × (− 0.2146) + TGF-β1 × (0.42174)] and were separated according to the median

^c^Multivariate Cox proportional regression was performed by all listed factors together

### Double-positive signals of M1 and M2 markers on MPE-Mφ could be maintained by cancer cell-derived conditioned medium (CM)

**Fig. 2:**
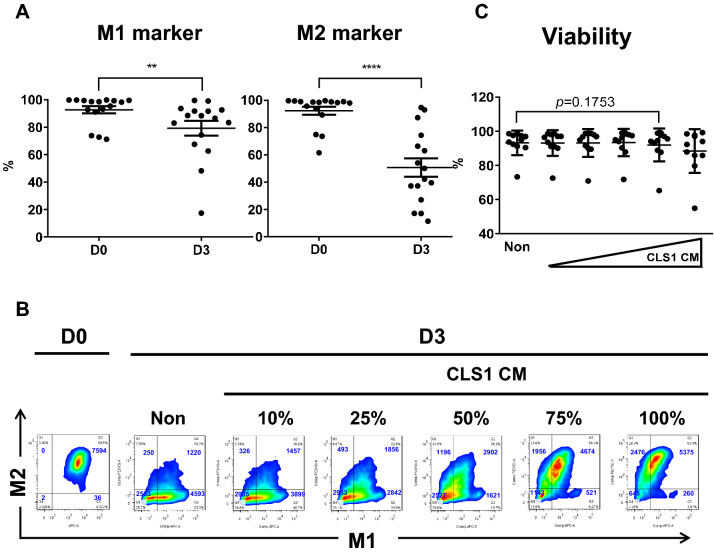

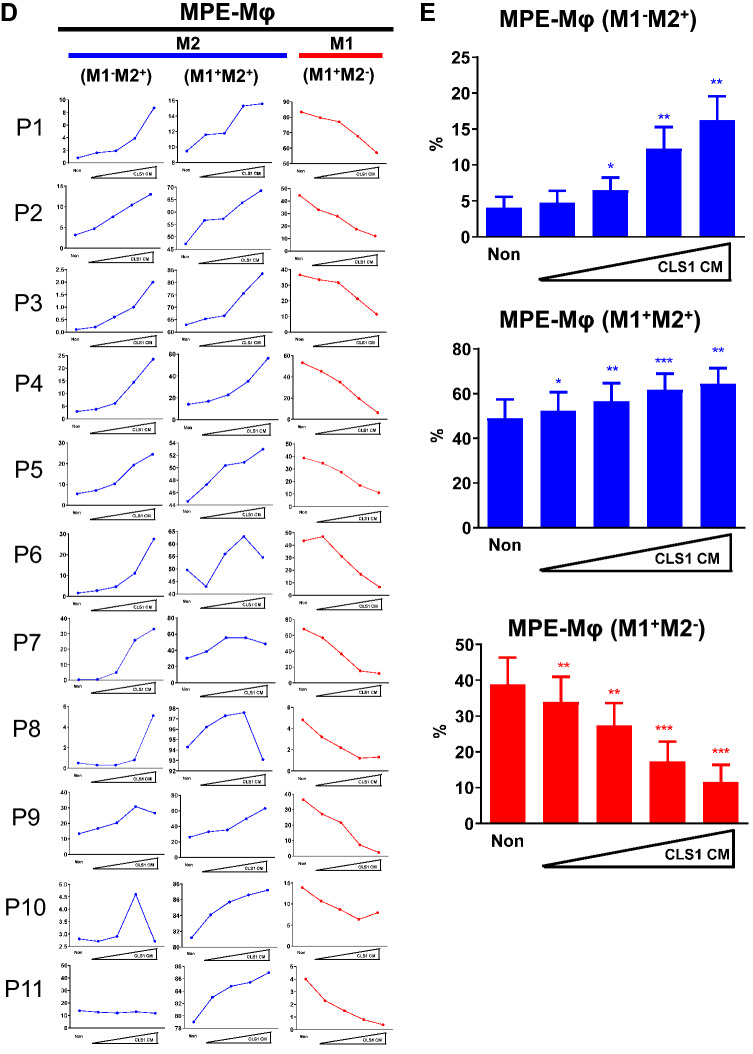

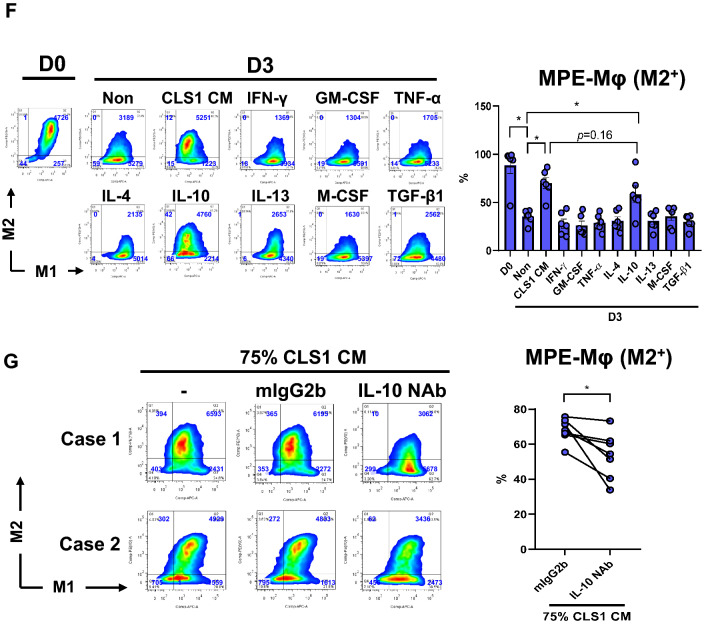
M1 and M2 marker expression of MPE-Mφ was maintained with CLS1 cell-derived conditioned medium (CLS1 CM). **a** M1 and M2 marker expression of MPE-Mφ decreased on day 3. Validation of the expression of M1 (HLA-DR) and M2 markers (CD163) by MPE-Mφ on day 0 and day 3 by flow cytometry with 16 MPE samples. Day 0 is shown as D0, while day 3 is shown as D3 in the figure. D3 groups of MPE-Mφ were maintained in RPMI medium with 10% fetal bovine serum (FBS) for 3 days. Data shown as the means ± SEM and compared using a paired *t* test; **p < 0.01; ****p < 0.0001. One point indicates 1 MPE-Mφ case. *N* = 16. **b** MPE-Mφ were cocultured with increasing concentrations (10–100%) of CLS1 cell-derived conditioned medium (CLS1 CM) to maintain the double-positive M1 and M2 marker signals until day 3. Day 0 is shown as D0, while day 3 is shown as D3 in the figure. Blue words in the figures indicate the number of live macrophages. **c** Determination of the viability (%) of MPE-Mφ after coculturing with CLS1 CM for 3 days. Data are presented as the means ± SEM and compared using a paired *t* test. *N* = 11. **d** Representation of M2 (M1^−^M2^+^ and M1^+^M2^+^, blue line) and M1 (M1^+^M2^−^, red line) expression patterns with CLS1 CM (10%, 25%, 50% and 75%) among 11 MPE-Mφ samples. **e** Evaluation of M2 (blue bar) and M1 (red bar) expression % among 11 MPE-Mφ samples cultured with CM (10%, 25%, 50% and 75%). Data are expressed as the means ± SEM and compared using a paired *t* test; *p < 0.05; **p < 0.01; ***p < 0.001. A * above the bar indicates that a comparison between the indicated group and the non-group. **b**–**e** The non-group indicates MPE-Mφ that were cultured in RPMI medium with 10% FBS, and all other groups were cultured in the indicated medium with 10% FBS. **f** Expression of M1 (HLA-DR) and M2 (CD163) markers with different cytokine stimulation was detected by flow cytometry at day 3. All groups, unless specifically mentioned, were cultured in control medium (RPMI medium with 10% FBS). All cytokines in this study were used at a concentration of 100 ng/ml. Day 0 is shown as D0, while day 3 is shown as D3 in the figure. Blue words in the figures indicate the number of live macrophages (left panel). Flow cytometry was used to identify the M2^+^ populations among 6 MPE-Mφ samples with different treatments. One blue circle indicates one MPE-Mφ sample. Data shown as the means ± SEM and compared using a paired *t* test; *p < 0.05, *N* = 6 (right panel). **g** Determination of the % of M2^+^ MPE-Mφ after culturing with CLS1 CM in the presence of anti-IL-10 neutralizing antibody (left panel). The 75% CLS1 CM supplemented with 10% FBS was preincubated with IL-10 neutralizing antibody (NAb) or isotype-matched antibody (mIgG2b) for 1 h and then added to MPE-Mφ for 2 days. M1 (HLA-DR) and M2 (CD163) markers were validated by flow cytometry. The concentration of isotype-matched antibody (mIgG2b) and IL-10 NAb was 10 μg/ml. Blue words in the figures indicate the number of live macrophages. Case 1 and case 2 were two independent MPE-Mφ subjects (left panel). Flow cytometry was used to identify the M2^+^ populations among 7 MPE-Mφ samples with different treatments as described in the left panel. One blue circle indicates one MPE-Mφ sample. Data shown as the means ± SEM and compared using a paired t test; *p < 0.05, *N* = 7 (right panel)

The MPE-Mφ showed plasticity in being able to transition to M1 or M2 macrophages. Of interest, we found that all of the isolated MPE-Mφ were double-positive for M1 (HLA-DR) and M2 (CD163) markers on day 0, but their signals decreased on day 3, especially the CD163 marker (*p* < 0.01 for HLA-DR; *p* < 0.0001 for CD163) (Fig. 2a). These results indicated that MPE-Mφ may lose polarity after removal from MPE conditions. To maintain the polarity of MPE-Mφ in vitro, CLS1 cancer cells-derived CM was used and showed concentration-dependently increasing the level of HLA-DR/CD163 expression, and expression plateaued at 75% CM (Fig. 2b) with no cytotoxicity (*p* = 0.1753, Fig. 2c). In addition, the M2 (M1^−^M2^+^, M1^+^M2^+^) and M1 signals (M1^+^M2^−^) in another 11 MPE-Mφ samples were also analyzed after culturing with increased concentrations of CM (Fig. 2d); whereas, the accumulated data showed in Fig. 2e, which indicated a significant increase in the M2 population (blue color) and a dramatic decrease in the M1 population (red color) with increased concentrations of CM. We further confirmed that the A549 CM could also maintain the M2-spectrum in vitro; but not cell-free MPE, which may cause significant cytotoxicity (Supplementary Fig. 5 & Supplementary Results).

Several cytokines enriched in the CM might be responsible for M2 polarization. To identify the key soluble factors of CM on maintenance of the MPE-Mφ pattern, we examined the effects the cytokines (IFN-γ, GM-CSF, TNF-α, IL-4, IL-10, IL-13, M-CSF, and TGF-β1), which has been reported and may enrich in CLS1 CM [23], on MPE-Mφ differentiation. In Fig. 2f, we showed that IL-10 could significantly increase the M2^+^ population compared to control medium (58.6 ± 8.59% vs. 35.31 ± 2.82%, N = 6, *p* < 0.05). In addition, blockade of IL-10 in the CLS1 CM using IL-10 neutralizing antibody could significantly decrease the CLS1 CM-induced M2 population in MPE-Mφ, compared to the isotype control (67.93 ± 2.54% vs. 53.64 ± 5.04%, *N* = 7, *p* < 0.05) (Fig. 2g). Our data further pointed out that IL-10 could be the key soluble factor in the CLS1 CM maintaining MPE-Mφ in a M2-like state.

### Combination of IFN-γ and β-glucan can repolarize MPE-Mφ into M1 macrophages

**Fig. 3:**
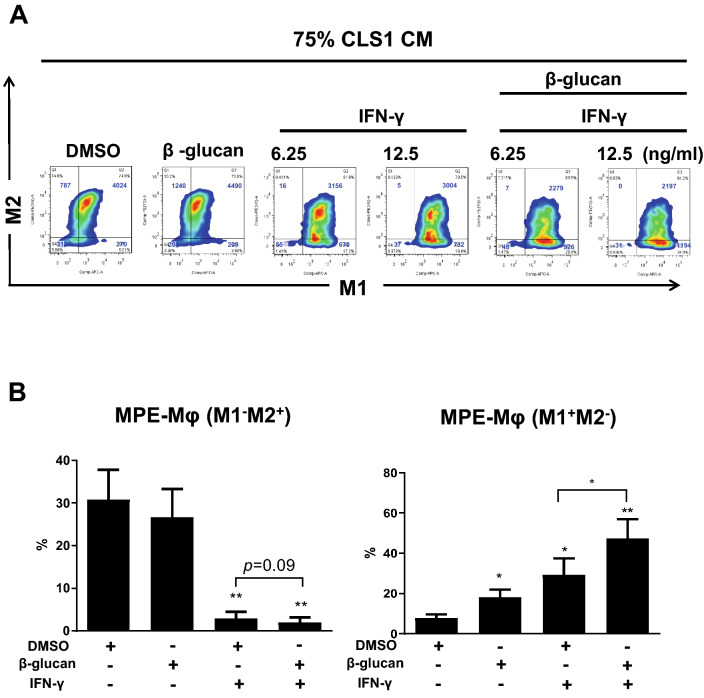
The combination of IFN-γ and β-glucan repolarized MPE-Mφ from M2 to M1 Mφ. **a** Expression of M1 (HLA-DR) and M2 (CD163) markers with different treatments, as indicated in the figure, was determined by flow cytometry. All groups were cultured in 75% CLS1 CM with 10% FBS until day 3. The concentration of β-glucan was 0.3 μg/ml. Blue words in the figures indicate the number of live macrophages. **b** Flow cytometry was used to identify M2 (M1^−^M2^+^, left panel) and M1 (M1^+^M2^−^, right panel) macrophages among 10 MPE-Mφ samples with different treatments. Data are expressed as the means ± SEM and compared using a paired t test; *p < 0.05; **p < 0.01. A * above the bar indicates that a comparison between the indicated group and the DMSO group. All groups were cultured in 75% CLS1 CM with 10% FBS until day 3. The concentrations of β-glucan and IFN-γ were 0.3 μg/ml and 12.5 ng/ml, respectively. **a**, **b** DMSO was the vehicle control for β-glucan

To our knowledge, M1 macrophages can be stimulated via IFN-γ and toll-like receptor (TLR) ligands, and they can then perform specific roles in innate immune responses by producing specific immune-stimulating cytokines and exhibiting anti-tumor activity. Here, we proposed the repolarization of MPE-Mφ from M2 to M1 macrophages to improve anti-cancer therapies. IFN-γ increased the M1 signal (M1^+^M2^−^) from 5% to 17.2% (6.25 ng/ml of IFN-γ) or 19.4% (12.5 ng/ml of IFN-γ), while β-glucan did not change the M1 percentage (M1^+^M2^−^) (5–3.4%). Compared to IFN-γ alone, IFN-γ synergistically working with β-glucan significantly enhanced the M1 signal (M1^+^M2^−^) from 5 to 28.4% or 34.9% (Fig. 3a). To validate this finding, 10 MPE-Mφ samples were collected and treated with IFN-γ, β-glucan or a combination of the two in 75% CLS1 CM. We confirmed that IFN-γ alone could lower the M2 population (M1^−^M2^+^) (*p* < 0.01) (Fig. 3b, left panel) and tended to increase M1 macrophage percentages (M1^+^M2^−^) (*p* < 0.5) (Fig. 3b, right panel). Of most importance, combined treatment with β-glucan and IFN-γ achieved the most significant increase in the M1 macrophage population (from 10 to 50%) compared to those that were treated with DMSO only (*p* < 0.01) (Fig. 3b, right panel).

### Reversing the high-risk to low-risk signature and improving anti-cancer activity by repolarizing M2 macrophages to M1 macrophages using a combined IFN-γ and β-glucan treatment

**Fig. 4:**
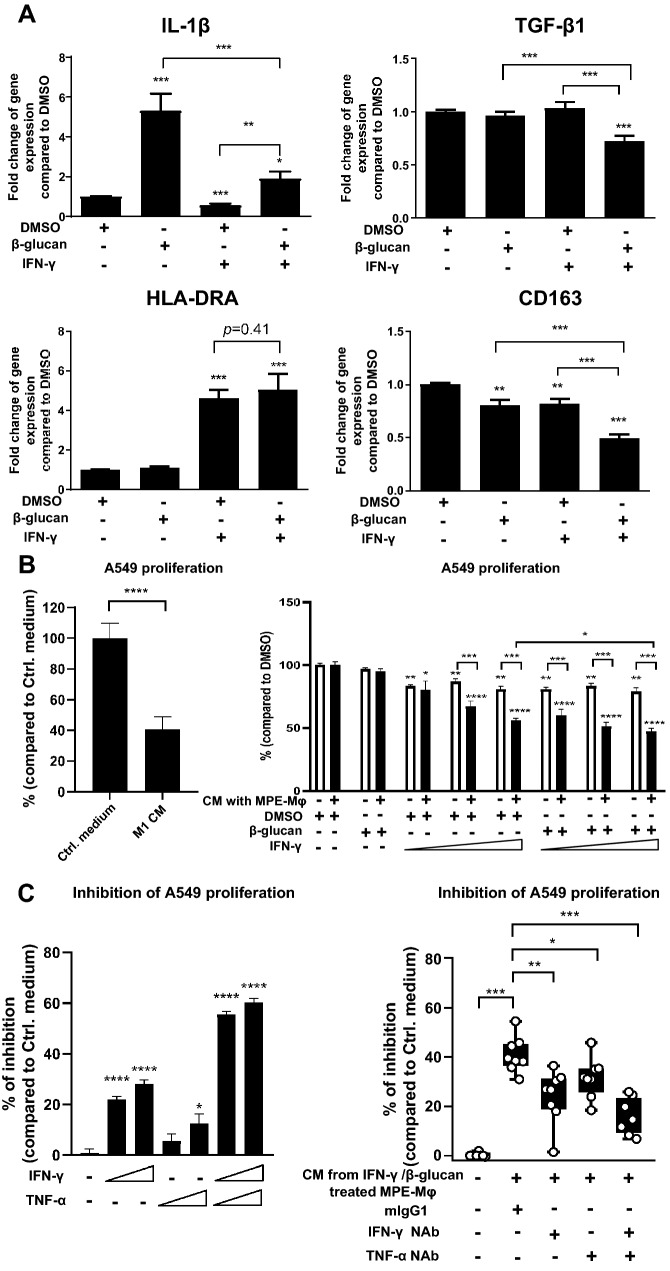
Combination of IFN-γ and β-glucan repolarized MPE-Mφ toward the indicated M1 (IL-1β and HLA-DRA) and M2 (TGF-β1 and CD163) gene signatures and inhibited lung cancer cell proliferation. **a** Validation of M1 (IL-1β/HLA-DRA) and M2 (TGF-β1/CD163) gene expression after stimulation with the indicated treatments. MPE-Mφ were stimulated with the indicated treatment as described under the bar. Total RNA was harvested at 18 h post-stimulation for reverse-transcription into complementary DNA (cDNA). Gene expression levels were determined by real-time PCR and normalized to TATA-Box Binding Protein (TBP). Fold changes in gene expression on the *y*-axis were calculated based on the expression of the same gene in the DMSO-treated group. Data shown as the means ± SEM and compared using a paired t test; **p < 0.01; ***p < 0.001; *N* = 5. A * above the bar indicates a comparison between the indicated group and the DMSO group. The concentrations of β-glucan and IFN-γ were 0.3 μg/ml and 3.125 ng/ml, respectively. All groups were cultured in 75% CLS1 CM with 10% FBS. **b** Validation of the anti-proliferative effect of coculturing with CM derived from THP-1-derived M1 macrophages (positive control, left panel) and CM with the indicated treatments (right panel). Anti-tumor effects are presented as the percentage of control medium (Ctrl. medium) (left panel) and DMSO group (right panel). Data are expressed as the means ± SEM and were compared using the Mann–Whitney *U* test; *p < 0.05; **p < 0.01; ***p < 0.001; ****p < 0.0001, *N* = 3. Ctrl. Medium indicates RPMI medium supplemented with 10% FBS. The concentration of β-glucan was 0.3 μg/ml and IFN-γ was 3.125 ng/ml, 6.25 ng/ml, or 12.5 ng/ml. **a**, **b** DMSO was the vehicle control for β-glucan. **c** Validation of the anti-proliferative effect contributed by IFN-γ/β-glucan-treated MPE-Mφ. A549 cells were stimulated with IFN-γ (100 ng/ml), TNF-α (100 ng/ml) or both cytokines for 3 days. Inhibition of A549 proliferation is shown as percentages of inhibition over control medium (all marks were -) (*N* = 3, left panel). CM from IFN-γ/β-glucan-treated MPE-Mφ was preincubated with IFN-γ, TNF-α or both neutralizing antibodies (NAb) (10 μg/ml) for 1 h and then added to A549 cells. Inhibition of A549 proliferation is shown as percentages of inhibition over control medium (all marks were -). One circle indicates 1 MPE-Mφ case (*N* = 8, right panel). Data are expressed as the means ± SEM and were compared using the Mann–Whitney *U* test; *p < 0.05; **p < 0.01; ***p < 0.001; ****p < 0.0001. Control medium indicates RPMI medium supplemented with 10% FBS. The concentration of β-glucan was 0.3 μg/ml and IFN-γ was 12.5 ng/ml

In terms of the potential clinical applications of targeting MPE-Mφ, our data showed that MPE-Mφ could be repolarized via combined treatment with IFN-γ and β-glucan (Fig. 3). We examined whether the high-risk signature of M1 (IL-1β) and M2 (TGF-β1) macrophages could be reversed via IFN-γ and β-glucan. Compared to the DMSO-treated group, β-glucan alone increased IL-1β gene expression (*p* < 0.001) but had no impact on TGF-β1. IFN-γ alone decreased IL-1β expression (*p* < 0.001), but IFN-γ alone also had no effect on TGF-β1. The combination of IFN-γ and β-glucan reversed the IFN-γ downregulation of IL-1β and efficiently lowered TGF-β1 expression (Fig. 4a, upper panels). Co-treatment with IFN-γ and β-glucan did not increase HLA-DRA expression more than IFN-γ alone (*p* = 0.41, Fig. 4a, lower left panel), but significantly downregulated CD163 expression, more than IFN-γ or β-glucan alone (*p* < 0.001, Fig. 4a, lower right panel). The accumulated evidences indicated that M1 conditioned medium (M1 CM) could inhibit tumor growth via TNF-α, CXCL9, IFN-γ, or CCL3 in different types of cancers [27–29]. Here, we used M1 CM as a positive control and showed that the M1 CM could significantly inhibit tumor cell growth (*p* < 0.0001) (Fig. 4b, left panel). To investigate the anti-cancer effect of CM from IFN-γ/β-glucan-educated MPE-Mφ, A549 cells were co-cultured with the CM. The CM from IFN-γ/β-glucan-educated MPE-Mφ showed higher anti-cancer effect than the IFN-γ/β-glucan-treated control (47.08% vs. 78.93%, *p* < 0.001, when IFN-γ was at 12.5 ng/ml). Whereas, β-glucan- treated group, had a slight influence on cell proliferation (80.34%) (Fig. 4b, right panel).

**Fig. 5:**
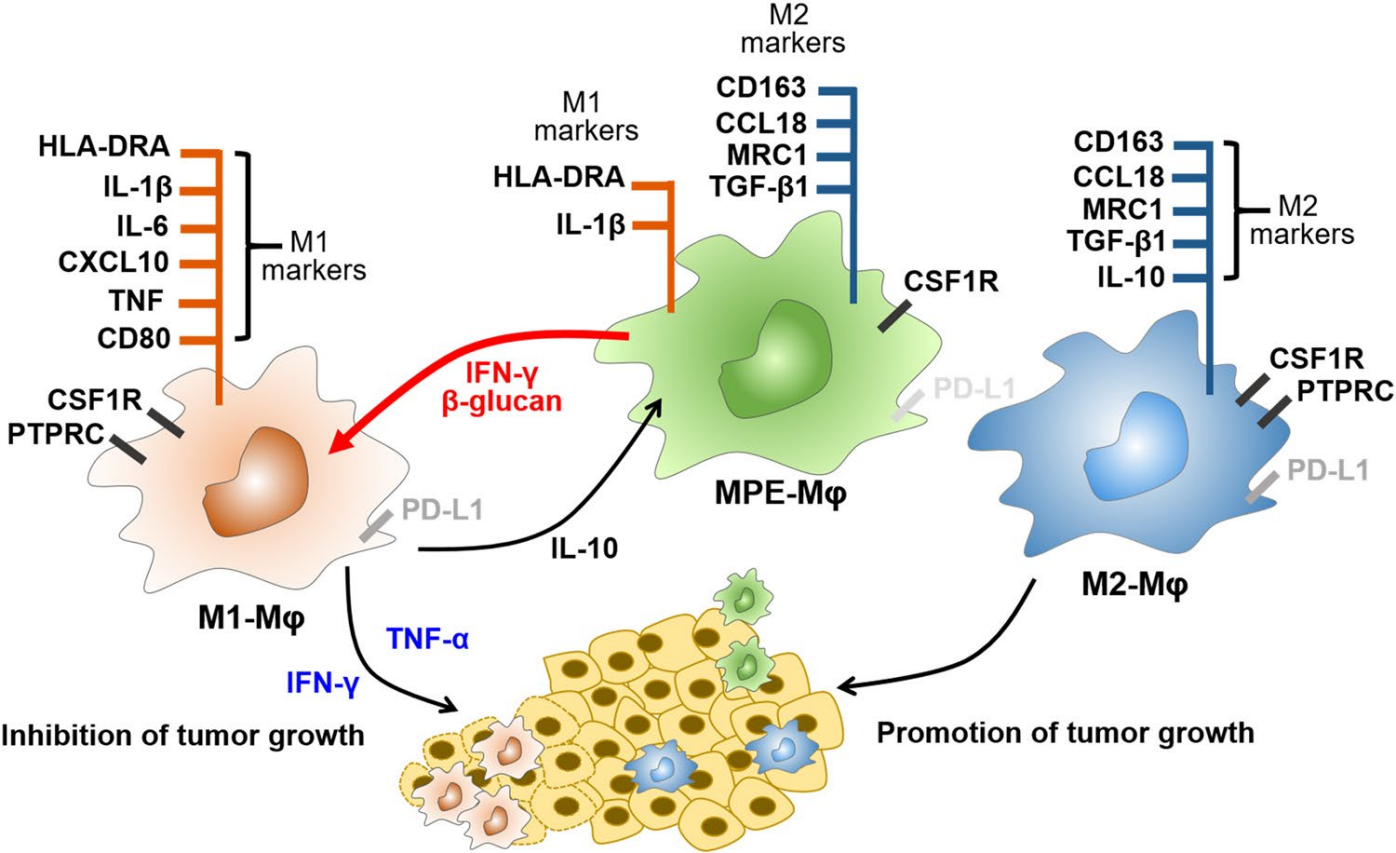
Characteristics of MPE-Mφ and IFN-γ/β-glucan-induced macrophage polarization. Figures represent M2–Mφ with M2 markers, M1–Mφ with M1 markers, and MPE-Mφ coexpressing M1 and M2 markers. IL-10 could maintain the MPE-Mφ phenotype. IFN-γ/β-glucan treatment re-educated MPE-Mφ to differentiate into tumor-killing macrophages with reduced M2 markers (TGF-β1 and CD163) and elevated IFN-γ and TNF-α production

To further identify the potential soluble factors from MPE-Mφ-derived M1 macrophages might inhibit cancer cell growth, we performed experiments to examine the effects of M1-related cytokines (TNF-α, CXCL9, IFN-γ, or CCL3) on A549 cell proliferation. Since CXCL9 and CCL3 are IFN-γ-related chemokines [30, 31], here we focused on studying the anti-cancer effects of IFN-γ and TNF-α. We found that recombinant IFN-γ could dose-dependently inhibit A549 cancer cell proliferation (21.9% vs. 28.1%), while TNF-α showed less effect (5.5% and 12.6%). By contrast, the combination of IFN-γ and TNF-α could improve the inhibitory effect (increased to 60.3% of growth inhibition, *p* < 0.0001) (Fig. 4c, left panel). This result indicated that both IFN-γ and TNF-α may contribute to the anti-tumor activity of MPE-Mφ-derived M1-like macrophages. Furthermore, using the anti-IFN-γ and anti-TNF-α neutralizing antibodies to block these soluble factors (IFN-γ or TNF-α) in the CM of the educated MPE-Mφ could reduce the anti-cancer effects, individually and synergistically (Fig. 4c, right panel). In addition, we also assessed the anti-cancer effects of CM from IFN-γ/β-glucan-treated MPE-Mφ in three other lung cancer cell lines (H1299, PC9 and CLS1). The anti-cancer effects of IFN-γ/β-glucan-treated MPE-Mφ, as well as M1 macrophages, could also be observed in the three other cell lines (Supplementary Fig. 6). These data raise the possibility that reversing the high-risk signature using IFN-γ/β-glucan may allow for an increase in the M1 macrophage population and enhance the anti-tumor efficiency of macrophages in MPE to improve anti-cancer therapy. These findings suggest that the MPE-Mφ with both M1 and M2 markers can be repolarized by IFN-γ and β-glucan through the activation of the JAK/STAT1 [32] and Syk-CARD9-ERK pathways [19] to downregulate the M2 markers TGF-β1 and CD163, thereby enhancing the repolarization of MPE-Mφ into tumor-inhibiting M1 macrophages (Fig. 5).

## Discussion

Macrophages around tumor cells (TAM) are associated with onco-immunity orchestration, tumor malignancy promotion, and metastasis [33]. M1/M2 polarization ratios and M1/M2 marker signatures have been suggested as potential prognostic factors to predict OS in lung cancer patients [13, 29], and the repolarization of TAM into M1 macrophages could be a novel strategy for anti-cancer therapy. In our study, we carried out a comprehensive analysis of the M1/M2/Mφ markers and the diversity of PD-L1 expression in MPE-Mφ, and identified a clinically relevant M1/M2 two-gene signature that can predict OS of NSCLC patients. Of most importance, we demonstrated that MPE-Mφ with a spectrum of M1/M2 phenotypes, such as a dual M1/M2 tendency, may predict clinical outcomes and potential plasticity. In addition, we reported for the first time that clinical-isolated MPE-Mφ could be “re-educated” and show a potential benefit for anti-cancer therapy.

Approximately 70% of TAMs were identified as the immunosuppressive M2 phenotype and reported to be polarized from M1 during tumor progression [12, 14]. Our study identified CD14^+^CD68^+^ MPE-Mφ with double-positive signals for M1 (HLA-DR) and M2 (CD163) markers. Th1 (IFN-γ) or Th2 (IL-4 and IL-10) cytokines responsible for M1 or M2 macrophage polarization were detected in malignant pleural effusions of lung cancer patients in a previous study [34]. We suggested that the coexistence of Th1 and Th2 cytokines in MPEs may lead to the dual expression of M1/M2 markers by MPE-Mφ. In our study, we further found that soluble IL-10 is necessary for MPE-Mφ to be retained in an M2-like stage. Previous reports of TAMs in paraffin-embedded NSCLC specimens showed that only a small portion (3%) of TAMs coexpressed M1 (HLA-DR) and M2 (CD163) markers as determined by IHC staining signals [14]. By contrast, our findings found that the MPE-Mφ may be different from the TAMs in lung tumor tissues and showed a dual tendency in their M1/M2 patterns, which is similar to the data for the TAMs in pancreatic ductal adenocarcinoma (PDAC) that expressed both M1 (HLA-DR, IL-1β and TNF-α) and M2 (CD163 and IL-10) markers [35]. This result may explain why the differential tumor microenvironment (TME) can lead to distinct macrophage phenotypes that contribute to tumor progression and may subsequently impact the immunity of the microenvironment. Furthermore, we validated another 6 M1 and 5 M2 genes in MPE-Mφ, showing that MPE-Mφ had both M1 and M2 macrophage gene expression patterns, which may increase the complexity and heterogeneity of macrophages in MPEs. In addition to the M1/M2 ratio, M1/M2 signatures have also been studied as indicators to predict patient OS [13, 29]. Here we proposed that a 2-gene signature of IL-1β (M1) and TGF-β1 (M2) could represent the features of MPE-Mφ and tumor tissues (TCGA dataset) [22] to indicate their significance in the OS of late-stage NSCLC patients. Our previous report [29] also demonstrated that the gene expression profiles between M1 and M2 macrophage-stimulated A549 cells are significantly different, and these M1/M2-stimulated gene signatures could be used to predict the clinical outcomes of NSCLC patients. Here, we were able to more precisely perform late stage survival predictions by using only the two-gene signature of the M1 (IL-1β) and M2 (TGF-β1) genes directly from MPE-Mφ or lung cancer tissues.

To re-educate M2 macrophages toward the M1 type, previous reports have used human monocyte-derived macrophages or mouse bone marrow-cultured macrophages to identify that IFN-γ and β-glucan are involved in polarizing the M1-like phenotype and inhibiting the M2 polarization pathway [19, 36, 37]. To the best of our knowledge, this is the first study to use the cotreatment of IFN-γ and β-glucan to repolarize clinical MPE-Mφ samples from late stage adenocarcinoma patients. Our data showed a synergistic effect between the IFN-γ and β-glucan-induced signaling pathways for macrophage reprogramming from M2 to anti-tumor M1, and for efficiently downregulating the expression of TGF-β1. Interestingly, β-glucan treatment might induce the expression of IL-1β in MPE-Mφ, whereas IFN-γ decreased the basal IL-1β expression. Cotreatment with IFN-γ and β-glucan partially reversed the effects of IFN-γ on IL-1β expression by twofold compared to the basal level, which is comparable with previous cases of DCs being repolarized from Th17 into Th1 cells [38]. As a result, the combination of IFN-γ and β-glucan treatment of MPE-Mφ may re-educate MPE-Mφ to become the anti-tumor M1 type and the combination more responsible for Th1 subtype differentiation in the tumor microenvironment compared to either single treatment alone.

PD-L1 on immune cells or tumor cells can interact with the co-inhibitory PD-1 receptor on T cells to attenuate T cell function [39], and PD-L1 expression can predict checkpoint inhibitor (anti-PD-L1) efficacy [40]. Our report found that most MPE-Mφ express relatively low levels of PD-L1, which may dampen anti-PD-L1 treatments, and this is correlated with previous clinical observations [41]. At the same time, the low PD-L1 expression group was correlated with higher expression levels of IL-10, which raises the possibility that MPE-Mφ may maintain low PD-L1 expression when they are in a resting M2-type state. IFN-γ and IL-1β signaling are involved in regulating PD-L1 expression [39, 42, 43]. Our results indicated that IFN-γ increased PD-L1 expression, while its combination with β-glucan elevated or caused a heterogeneous response in PD-L1 levels compared to IFN-γ alone among 6 different patients (Supplementary Fig. 7). Therefore, we suggested that targeting MPE-Mφ via IFN-γ and β-glucan may modulate the immunity of the tumor microenvironment by increasing the PD-L1 expression of MPE-Mφ for checkpoint inhibitor therapy and deceasing potential IL-10 secretion for M2 macrophages polarization; we propose that this can serve as an adjuvant for anti-cancer therapy to reduce tumor progression, metastasis, and drug resistance.

In summary, our data show that macrophages in MPEs have a heterogeneous M1/M2 gene expression pattern with an M2-like phenotype and diverse PD-L1 expression. Moreover, a 2-gene signature (IL-1β/TGF-β1) is correlated with the OS of lung cancer patients. We propose targeting MPE-Mφ by using IFN-γ and β-glucan to repolarize them into IL-1β-high/TGF-β-low M1 macrophages. Taken together, we conclude that M1/M2 macrophages may play various roles in lung cancer development. M1/M2 tendency and our two-gene signature may be used as biomarkers to predict patient OS. Re-educating MPE-Mφ toward the M1 type may represent an Immuno-Oncology (IO) adjuvant to improve anti-cancer treatment efficacy in the future.

### Electronic supplementary material

Below is the link to the electronic supplementary material.

Supplementary file1 (PDF 12611 kb)

## Abbreviations

MPE: Malignant pleural effusion
Mφ: Macrophage
NSCLC: Non-small cell lung cancer
OS: Overall survival
PD-L1: Programmed death-ligand 1
TCGA-LUAD: The Cancer Genome Atlas Lung Adenocarcinoma
