# Supplementary material for: The M1/M2 spectrum and plasticity of malignant pleural effusion-macrophage in advanced lung cancer
Source: Cancer Immunol Immunother. 2020 Nov 11;70(5):1435–50. doi: 10.1007/s00262-020-02781-8 (PMC8053174; doi:10.1007/s00262-020-02781-8)

**The M1/M2 Spectrum and Plasticity of Malignant Pleural Effusion-Macrophage in Advanced Lung Cancer**

Ming-Fang Wu, [Chih-An Lin](https://www.clinicalkey.com/#!/search/Lin%20Chih-An/%7B%22type%22:%22author%22%7D), Tzu-Hang Yuan, Hsiang-Yuan Yeh, Sheng-Fang Su, Chin-Ling Guo, Gee-Chen Chang, Ker-Chau Li, [Chao-Chi Ho](https://www.atsjournals.org/author/Ho%2C+Chao-Chi)* , Huei-Wen Chen*

1. **Supplementary Materials**

**1.1. Preparation of CLS1 cells-derived conditioned medium (CLS1 CM)**

On day 1, 3x10^6^ CLS1 isolated from our lab (1) were seeded in the 10 cm^2^ dish in 8 ml of complete medium [RPMI with 10% fetal bovine serum (FBS)]. On day 2, the complete medium was replaced with serum free medium (SFM) and cultured for another 48 h. The supernatants were collected as CLS1 CM.

**1.2. Preparation of THP-1-derived M1 macrophages CM (M1 CM)**

THP-1 (2x10^7^) were stimulated with phorbol 12-myristate 13-acetate (PMA) at 40 ng/ml for 24 h, followed by recovering with complete medium [RPMI with 10% fetal bovine serum (FBS)] for 24 h. After recovering, SFM was added and collected at 24 h and 48 h as THP-1 CM. THP-1 cells (5x10^6^) were further cultured with THP-1 CM for 6 days. The cells were recovered with complete medium [RPMI with 10% fetal bovine serum (FBS)] for 24 h (M0 macrophages). The M0 macrophages were stimulated with IFN-γ (20 ng/ml) for 16 h and further lipopolysaccharide (LPS) activation for 48 h (100 pg/ml). After recovering for 24 h, fresh complete medium [RPMI with 10% fetal bovine serum (FBS)] was added and was then collected at 24 h as a THP-1-derived M1 macrophages CM (M1 CM).

**1.3. RNA extraction and real-time polymerase chain reaction (PCR)**

Total cellular RNA isolated using Trizol reagent (Invitrogen Life technologies, CA, USA) was used as the template to synthesize the complementary DNA (cDNA) for PCR reaction using the SuperScript TM II First Strand cDNA Synthesis Kit (Invitrogen Life technologies, CA, USA). PCR was performed in the ABI PRISM 7900HT (Applied Biosystems) using the following procedure: Samples were denatured at 95℃ for 5 min, followed by 40 cycles of 95℃ for 15 s, 60℃ for 30 s and 72℃ for 30 s. Gene expression levels of each gene were calculated based on their respective internal control, TATA box binding protein (TBP). The primer sequences were shown in Supplementary Table 1.

**1.4 Co-culture of D0 malignant pleural effusion-Macrophages (MPE-Mφ) with increasing concentrations (10%-100%) of CLS1 CM**

The day 0 (D0) MPE-Mφ (5x10^5^/ml) were co-cultured in a 6-well ultra-low dish (Corning) with CLS1 CM: 10%, 25%, 50%, 75% and 100%, supplemented with 10% FBS. On day 3 (D3), the cells were scraped and assayed for M1 (HLA-DR) and M2 (CD163) markers.

**1.5 Co-treatment of IFN-γ and β-glucan in vitro**

The day 0 MPE-Mφ were seeded in a 6-well ultra-low dish (Corning) with 75% CLS1 CM supplemented with 10% FBS. The indicated concentrations of IFN-γ and β-glucan were added to the cell simultaneously. At the indicated time point, the cells were analyzed for the expression of 4 genes (IL-1β, TGF-β1, CD163 and HLA-DRA) (day 1), and the expression of M1 and M2 markers by flow cytometry (day 3); the supernatants were obtained after treatment (CM with MPE-Mφ “+” group) (day 2) for A549 proliferation assay.

**1.6. A549 proliferation assay**

A549 cells (1x10^3^) were seeded in a 96-well dish. At 24 h postseeding, the culture medium was removed, followed by adding the indicated CM or treatments. After 72 h, the viable cell numbers were assayed using a Cell Counting Kit-8, according to the manufacturer’s instructions (Dojindo Laboratories).

**1.7. Statistical analysis**

Survival analysis and presentation were based on R Language (2), [Kaplan-Meier methods](https://www.sciencedirect.com/topics/medicine-and-dentistry/kaplan-meier-method), and the [log-rank test](https://www.sciencedirect.com/topics/medicine-and-dentistry/log-rank-test). Gene expression levels of MPE-Mφ were calculated and normalized as described in “Materials and Method”. The Cancer Genome Atlas Lung Adenocarcinoma (TCGA-LUAD) samples were selected and normalized as described in (3) (4). For survival analysis, the R-package “My. stepwise”, version 0.1.0, published by Fu-Chang Hu in 2017 (<https://CRAN.R-project.org/package=My.stepwise>), the Cox regression model and [Kaplan-Meier methods](https://www.sciencedirect.com/topics/medicine-and-dentistry/kaplan-meier-method) were used. For survival curves drawing, the R-package ‘survminer’, version 0.4.3, published by Alboukadel Kassambara and Marcin Kosinski in 2018, was used (<https://CRAN.R-project.org/package=survminer>). The univariate and multivariate Cox proportional model included age, sex, EGFR gene mutation, clinical treatment defined within one month before MPEs enrolled and risk of population defined by gene expression according to two gene signature models and separated according to the median. Statistical analysis was performed using SPSS software, version 19.0 (Chicago, USA). A *p*value less than .05 was considered as a statistically significant difference.

1. **Supplementary Results**

**Supplementary Figure 5. M1 and M2 marker expression of MPE-Mφ was maintained with lung cancer cells-derived conditioned medium (CM), but not MPE.** In addition, we tried to use cell-free MPEs and conditioned media from A549 cells (A549 CM) to maintain M1 and M2 markers in a further 8 MPE samples (see Supplementary Figure 5). Our results indicated that the cell-free MPEs could not maintain the cell survival of the macrophages *in vitro* compared to the CLS1 CM and A549 CM (19.4 % viability rate for cell-free MPE vs. 80.2% viability rate for CLS1 CM and A549 CM on day3) (Supplementary Figure 5B). We found that A549 CM could also increase the percentages of M2 signals (M1^-^M2^+^ and M1^+^M2^+^, blue color) and decrease the percentages of M1 signals (M1^+^M2^-^, red color), and these changes were comparable to that of CLS1 CM (Supplementary Figure 5C). These results indicated the potential plasticity of MPE-Mφ and showed that cancer cell CM, but not cell-free MPE, could mimic the *in vivo* microenvironment of the MPE to maintain the M2-like spectrum.

1. **Supplementary References**

1. Chen WJ, Ho CC, Chang YL et al. (2014) Cancer-associated fibroblasts regulate the plasticity of lung cancer stemness via paracrine signalling. Nat Commun. 5: 3472. doi: 10.1038/ncomms4472

2. R Core Team (2013). R: A language and environment for statistical computing. R Foundation for Statistical Computing, Vienna, Austria. URL <http://www.R-project.org/>.

3. Grossman RL, Heath AP, Ferretti V, Varmus HE, Lowy DR, Kibbe WA, Staudt LM (2016) Toward a Shared Vision for Cancer Genomic Data. N Engl J Med. 375: 1109-12. doi: 10.1056/NEJMp1607591

4. Love MI, Huber W, Anders S (2014) Moderated estimation of fold change and dispersion for RNA-seq data with DESeq2. Genome Biol. 15: 550. doi: 10.1186/s13059-014-0550-8

5. Wei T, Simko V (2017) R package "corrplot": Visualization of a Correlation Matrix(Version 0.84). Available from <https://github.com/taiyun/corrplot>.

6. Kassambara A (2019) ggpubr: 'ggplot2' Based Publication Ready Plots. R package version 0.2.2.: <https://CRAN.R-project.org/package=ggpubr>.

## 7. Hadley W, Winston C, Lionel H et al (2019) ggplot2: Create Elegant Data Visualisations Using the Grammar of Graphics. R package version 3.2.1.: <https://cran.r-project.org/web/packages/ggplot2/index.html> (accessed date was on 2019/08/29)

1. **Supplementary Figures Legends**

**Supplementary Figure 1. Studying the M1/M2/Mφ genes expression of monocyte-derived macrophages (MDMs) from lung cancer patients and healthy controls (HCs) and malignant pleural effusion-macrophages (MPE-Mφ).** CD14^+^ monocytes isolated from peripheral blood mononuclear cells (PBMCs) of lung cancer patients and healthy controls were further cultured in complete RPMI 1640 medium (Hyclone) supplemented with 25 ng/mL human macrophage colony-stimulating factor (M-CSF) for 6 days at 37°C in 5% CO_2_. (N=5 for HC and patient). MPE-Mφ were isolated from MPEs by using anti-CD14 microbeads (N=60). Total RNA of macrophages were harvested for reverse-transcription into complementary DNA (cDNA). Fold changes in gene expression on the y-axis were determined by real-time PCR and normalized by TATA-Box Binding Protein (TBP). HC, healthy control; Patients, stage IV adenocarcinoma lung cancer patients. Data are expressed as the means ± SEM and compared using the Mann Whitney U test. **p*<0.05; ***p*<0.01; ****p*<0.001; *****p*<0.0001.

**Supplementary Figure 2. Visualizing correlations between M1/M2/Mφ genes and programmed death-ligand 1 (PD-L1).** Blue indicated the positive correlation while red indicated the negative correlation. No.1-14 performs the following genes: HLA-DRA, IL-1β, IL-6, CXCL10, TNF, CD80, CD163, CCL18, MRC1, TGF-β1, IL-10, CSF1R, PTPRC, PD-L1. All gene expression levels were calculated and normalized as described in “Materials and Methods”. Correlation figure was created using the R package corrplot (5)

**Supplementary Figure 3. Studying the correlation between TNF/CD80 and PD-L1 by constructing scatterplot with a linear regression.** (A-B) Two genes (TNF/CD80) expression negatively correlated with PD-L1 were selected from Supplementary Figure 2 and a scatterplot with PD-L1 was generated using R package ggpubr and ggplot2 (6)(7). The correlation (R) and *p* value are shown in the figure. One dot indicates an individual subject (N=59, no. 18 listed in Figure 1C was ruled out because of the undetectable PD-L1 expression).

**Supplementary Figure 4. The correlation between PD*-*L1 and M1/M2/Mφ markers gene expression**. The gene expression of M1/M2/Mφ markers in PD-L1 high and low populations. PD-L1 gene expression levels of MPE-Mφ subjects were divided into PD-L1 high and low populations as shown in Figure 1E (N=59, no. 18 listed in Figure 1C was ruled out because of the undetectable PD-L1 expression). H, PD-L1 high population (N=37); L, PD-L1 low population (N=22). Data shown as the means ± SEM and compared using an unpaired t test. Gene expression levels of MPE-Mφ genes were calculated and normalized as described in “Materials and Methods”

**Supplementary Figure 5. M1 and M2 marker expression of MPE-Mφ was maintained with lung cancer cells-derived conditioned medium (CM), but not MPE.** (A) MPE-Mφ were cultured with CLS1 cell-derived conditioned medium (CLS1 CM) or A549 CM or MPE to study the double-positive signals of M1 and M2 markers on day 3. Day 0 is shown as D0 while day 3 is shown as D3 in the figure. P1-P8 indicated 8 independent MPE-Mφ samples. NA means less viable cell (<10%) for the patient. (B) Determination of the viability (%) of MPE-Mφ after coculturing with CLS1 CM, A549 CM or MPE for 3 days. Data shown as the means ± SEM and compared using a paired t test; ****p*<0.001, N=8 (C) Representation of M2 (M1^-^M2^+^ and M1^+^M2^+^, blue bar) and M1 (M1^+^M2^-^, red bar) expression patterns with CLS1 CM, A549 CM or MPE among 8 MPE-Mφ samples. MPE group contains only the data from P2, P4-P6. Data shown as the means ± SEM and compared using a paired t test; **p*<0.05; ***p*<0.01. (A-C) Non group indicated that MPE-Mφ were cultured in RPMI medium; CLS1 CM indicated 75% CLS1 CM; A549 CM indicated 75% A549 CM; MPE indicated cell-free MPE. All groups were cultured in the indicated medium with 10% fetal bovine serum (FBS). A * above the bar indicates that a comparison between the indicated group and the non group.

**Supplementary Figure 6. Studying the anti-tumor effect by the combination of IFN-γ and β-glucan on different lung cancer cell lines (A)** Validation of the anti-proliferative effect of coculturing with CM derived from THP-1-derived M1 macrophages on three lung cancer cell lines (H1299, PC9, CLS1). Anti-tumor effects are presented as the percentage of control medium (Ctrl. Medium). Data were expressed as the means ± SEM and were compared using the Mann Whitney U test; *****p*<0.0001, N=3. Control medium (Ctrl. Medium) indicated RPMI medium supplemented with 10% FBS. (B) Validation of the anti-proliferative effect of coculturing MPE-Mφ CM with the indicated treatments on three lung cancer cell lines (H1299, PC9, CLS1). Anti-tumor effects are presented as the percentage of DMSO group. Data shown as the means ± SEM and compared using the Mann Whitney U test; **p*<0.05; ***p*<0.01; ****p*<0.001; *****p*<0.0001, N=3. A * above the bar indicates that a comparison between the indicated group and the DMSO group. The concentrations for β-glucan and IFN-γ was 0.3 μg/ml and 12.5 ng/ml, respectively. DMSO was the vehicle control for β-glucan.

**Supplementary Figure 7. Studying the PD-L1 expression with different treatments.** MPE-Mφ were stimulated with the indicated treatments as described under the bar. Total RNA were harvested at 18 h poststimulation for reverse-transcription into cDNA. Gene expression levels were determined by real-time PCR and normalized by TBP. Fold changes in gene expression on the y-axis were calculated based on the expression of the same gene in the DMSO-treated group. Patient 1-6 indicated 6 independent MPE-Mφ subjects, Data shown as the means ± SEM and compared using the Mann Whitney U test; **p*<0.05; triplicate. The concentrations of β-glucan and IFN-γ were 0.3 μg/ml and 3.125 ng/ml, respectively. All groups were cultured in 75% CLS1 CM with 10% FBS. DMSO was the vehicle control for β-glucan.

1. **Supplementary Tables**

**Supplementary Table 1. Primer sequences used in this study**

| **Primer** | **Forward** | **Reverse** |
| --- | --- | --- |
| CCL18 | GCTCTGCTGCCTCGTCTATACC | CCGGCCTCTCTTGGTTAGG |
| CD80 | GTGTTATCCACGTGACCAAGGA | TGCCAGTAGATGCGAGTTTGTG |
| CD163 | CAGTGCAGAAAACCCCACAA | AAAGGATGACTGACGGGATGA |
| CSF1R | AACAACACCAAGCTCGCAATC | CGGCATGTTGGAAATCTACTTGA |
| CXCL10 | ATTCCTGCAAGCCAATTTTGTC | CATCTCTTCTCACCCTTCTTTTTCA |
| HLA-DRA | AGCTGTGGACAAAGCCAACCT | AGGGCTGTTTGTGAGCACAGT |
| IL-1β | CAGTGGCAATGAGGATGACTTG | AGTGGTGGTCGGAGATTCGT |
| IL-6 | GACAGCCACTCACCTCTTCAGA | GTGCCTCTTTGCTGCTTTCAC |
| IL-10 | GAGGCTACGGCGCTGTCA | TCCACGGCCTTGCTCTTG |
| MRC1 | GAGGGAATCTGGTCTCCATACAA | AAGTGGAGTCCTTCATGTGATAGGT |
| PD-L1 | GAGGATATTTGCTGTCTTTATATTCATGA | GCTACCATACTCTACCACATATAGGTCCTT |
| PTPRC | CTACAGACCCAGTTTCCCCATT | TTCGCTGTGATGGTGGTGTT |
| TBP | ACGCCAGCTTCGGAGAGTT | CCTCATGATTACCGCAGCAAA |
| TGF-β1 | CGCGTGCTAATGGTGGAAA | TGTGTGTACTCTGCTTGAACTTGT |
| TNF | CTTCTGCCTGCTGCACTTTG | GGCCAGAGGGCTGATTAGAGA |
|  |  |  |

**Supplementary Table 2. Cox coefficient and *p* value of IL-1β and TGF-β1 genes for malignant pleural effusion-macrophage (MPE-Mφ) and the Cancer Genome Atlas Lung Adenocarcinoma (TCGA-LUAD) dataset**

| **MPE-Mφ^a^** | **Cox coefficient** | **H.R. ^c^** | **95% C.I. ^c^** | | ***p* value** |
| --- | --- | --- | --- | --- | --- |
| IL-1β | -0.21461 | 0.8069 | 0.6862 | 0.9488 | 0.00943 |
| TGF-β1 | 0.42174 | 1.5246 | 1.0706 | 2.1711 | 0.01937 |

| **TCGA ^b^** | **Cox coefficient** | **H.R.** | **95% C.I.** | | ***p* value** |
| --- | --- | --- | --- | --- | --- |
| IL-1β | -0.3146 | 0.7301 | 0.549 | 0.9709 | 0.0306 |
| TGF-β1 | 0.1633 | 1.1774 | 1.027 | 1.3492 | 0.0188 |

^a^ The risk score of gene signature for MPE-Mφ = (-0.21461 × expression level of IL-1β) + (0.42174× expression level of TGF-β1)

^b^ The risk score of gene signature for TCGA dataset = (-0.3146× expression level of IL-1β) + (0.1633 × expression level of TGF-β1)

^c^ C.I., confidence interval, H.R., hazard ratio

1. **Supplementary Figures**

**Supplementary Figure 1**

**
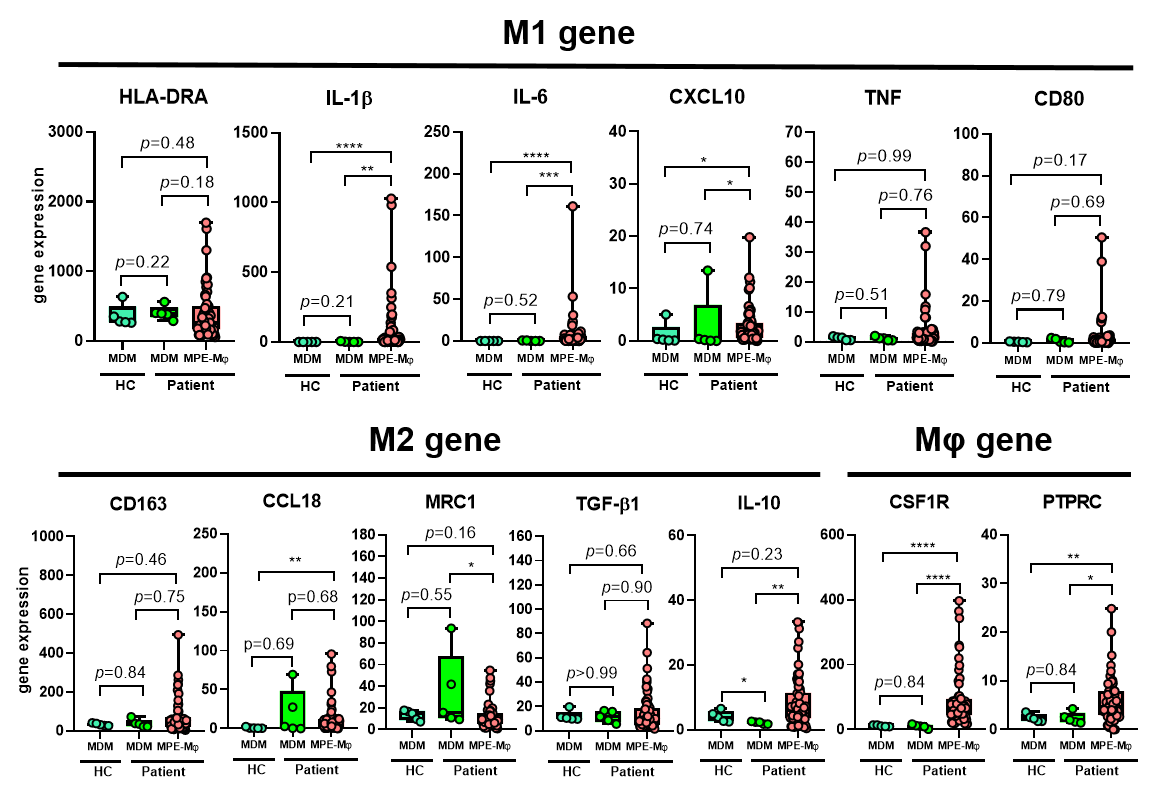
**

**Supplementary Figure 2**


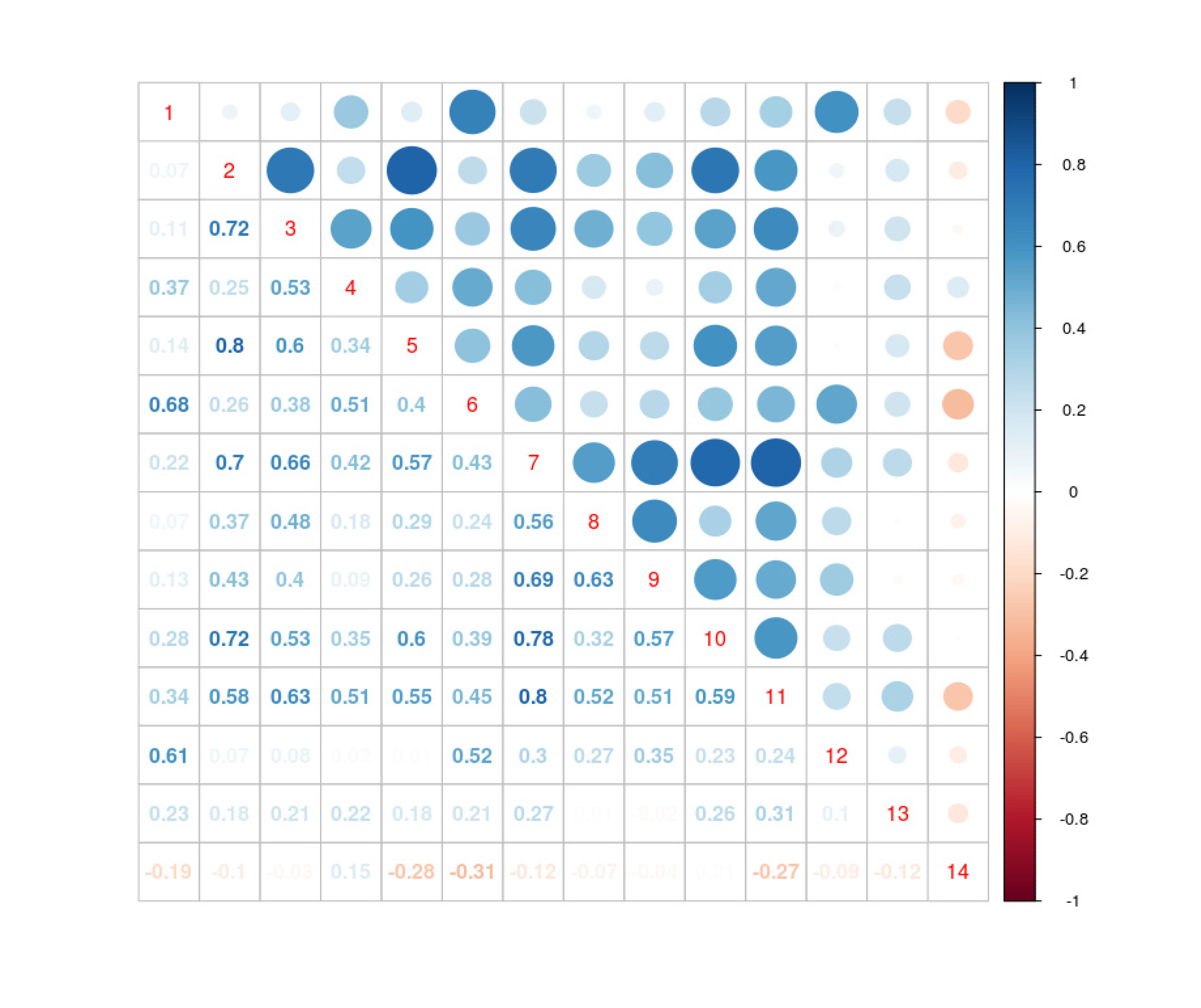


**Supplementary Figure 3**


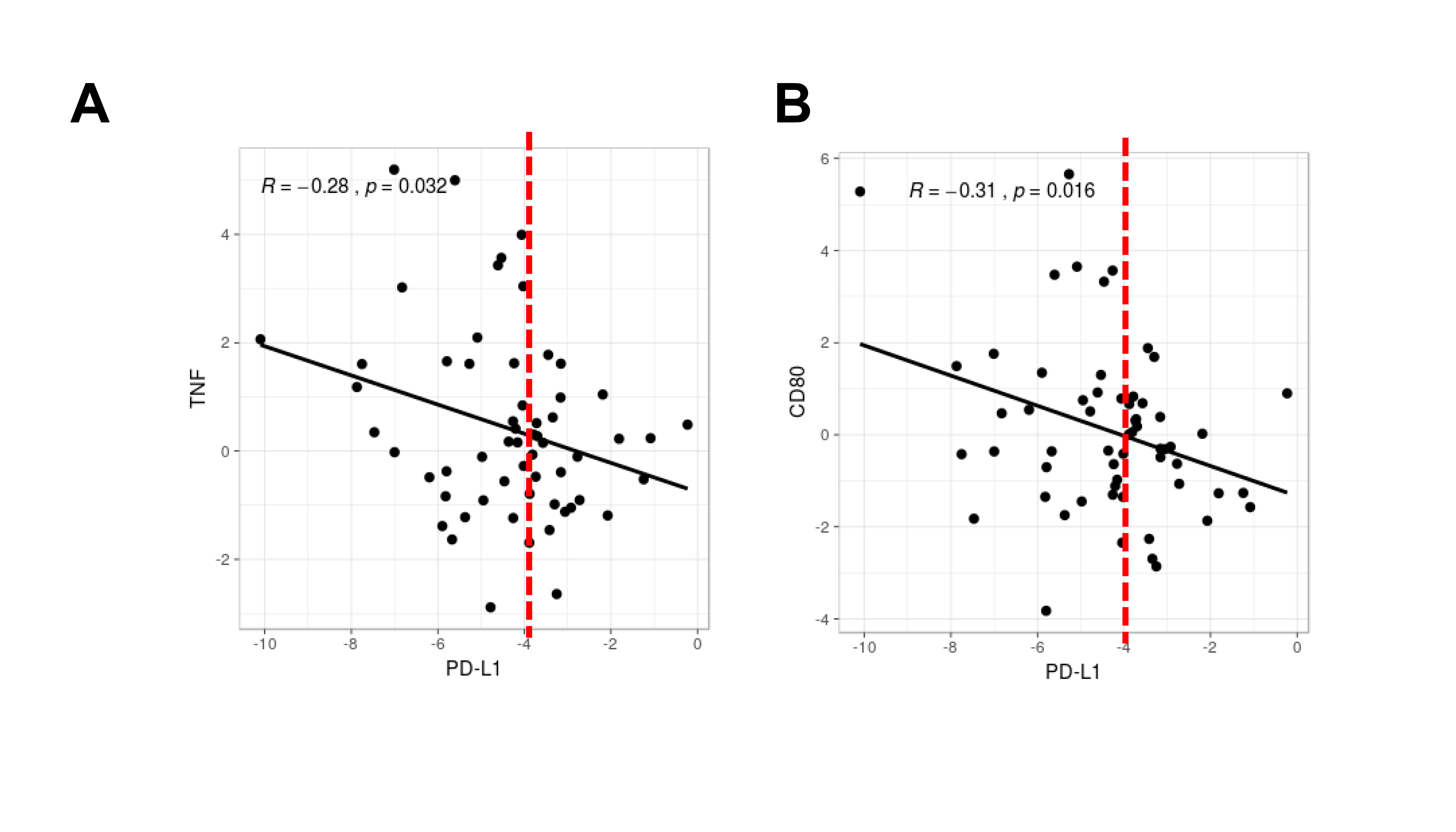


**Supplementary Figure 4**


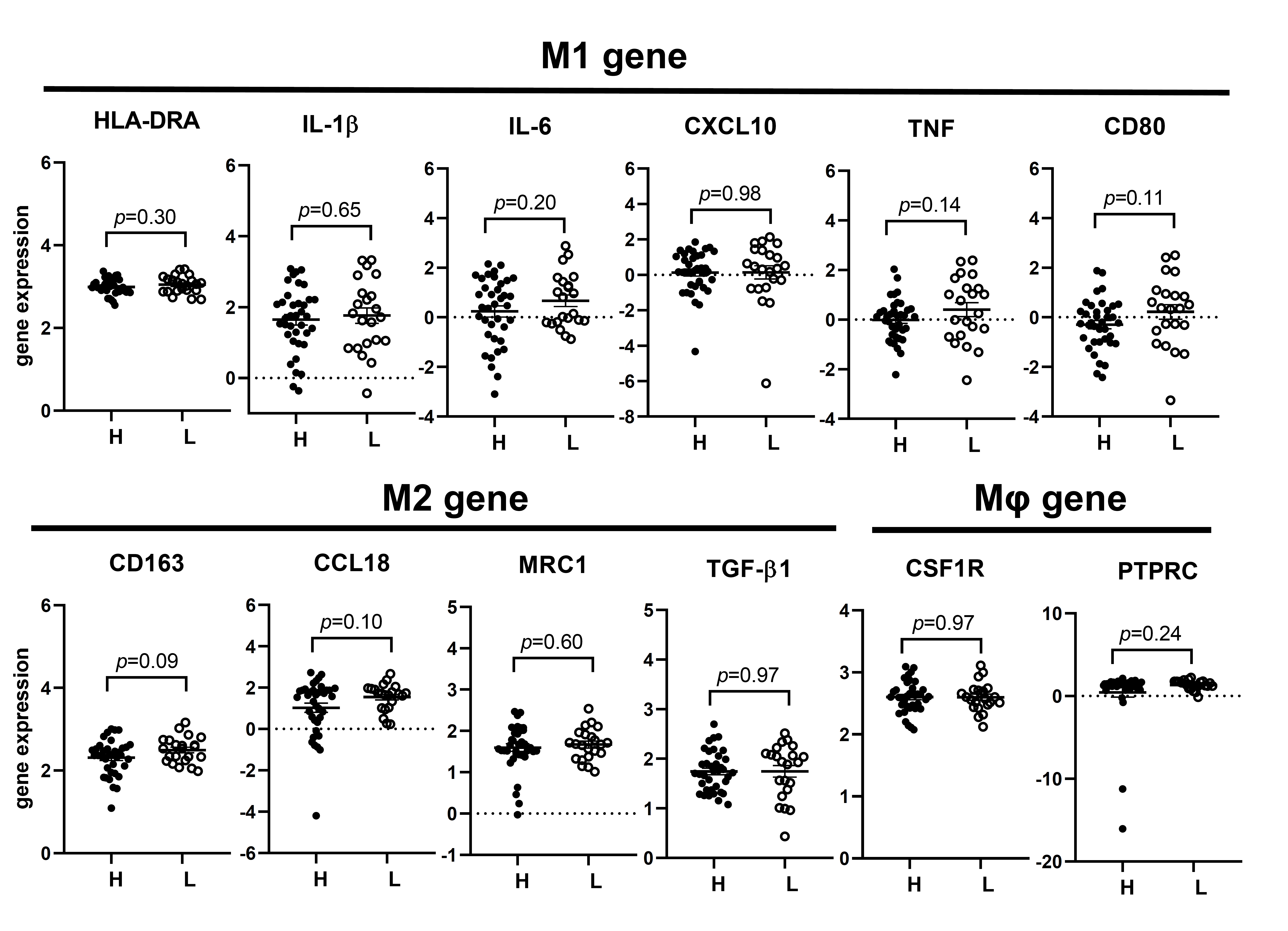


**Supplementary Figure 5**


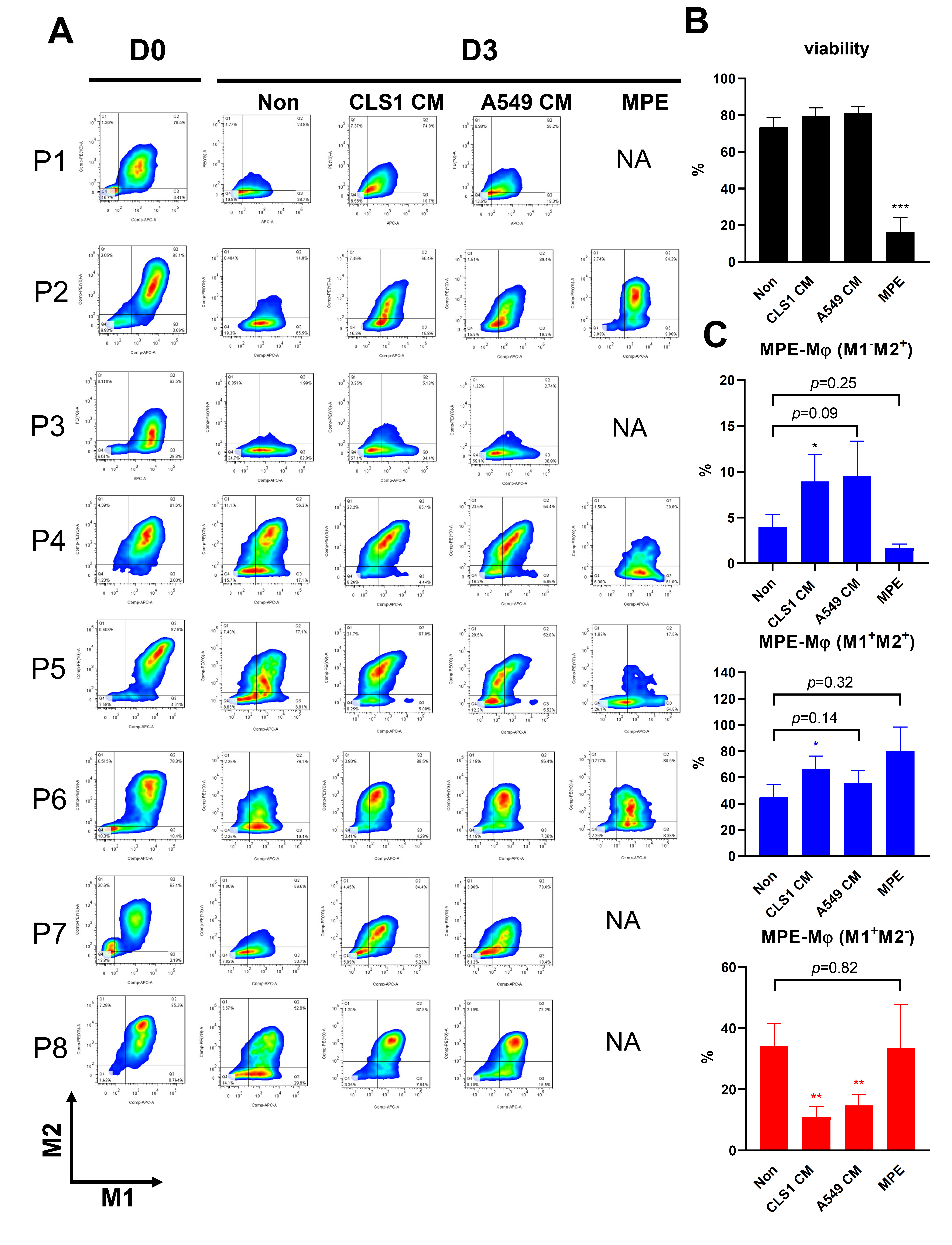


**Supplementary Figure 6**


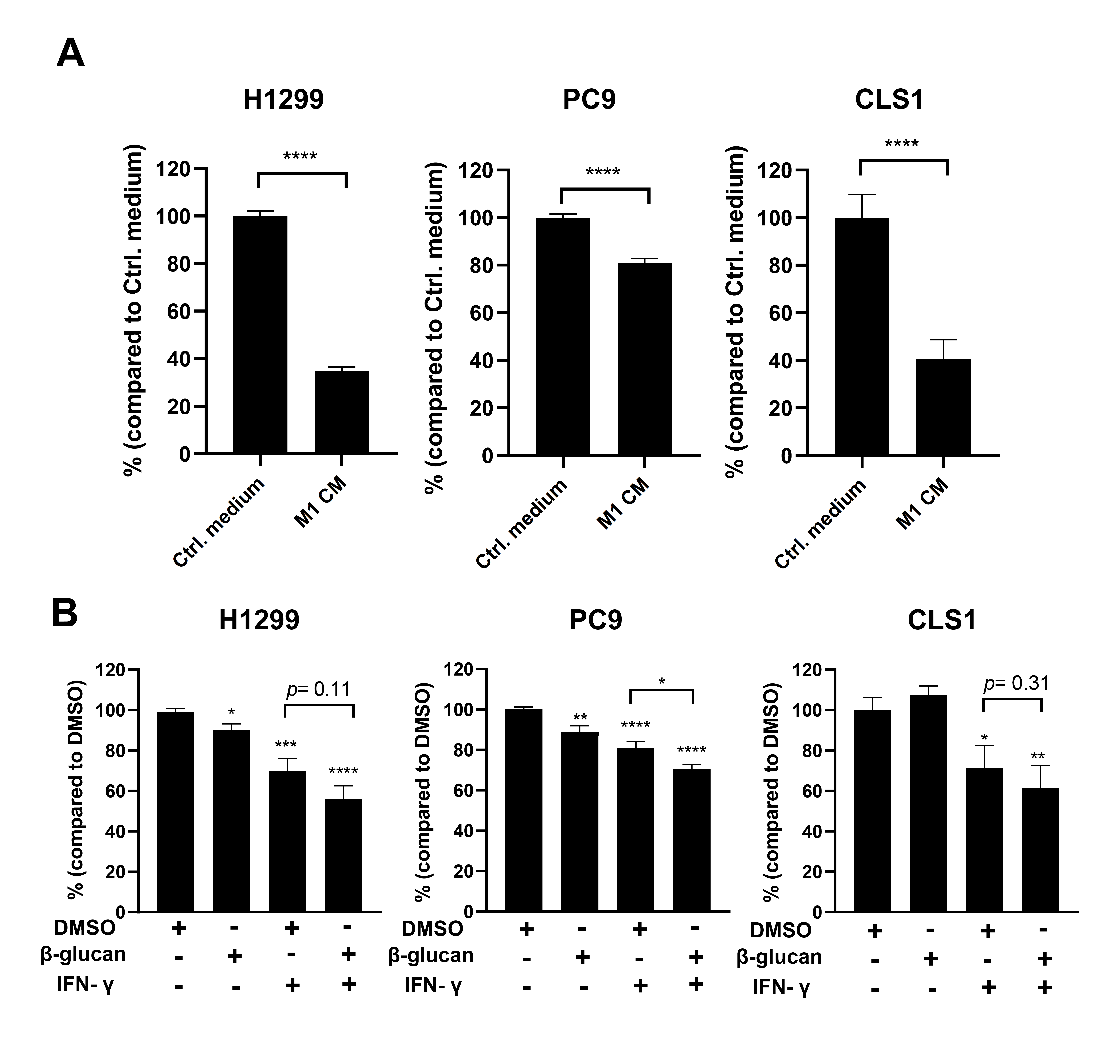


**Supplementary Figure 7**


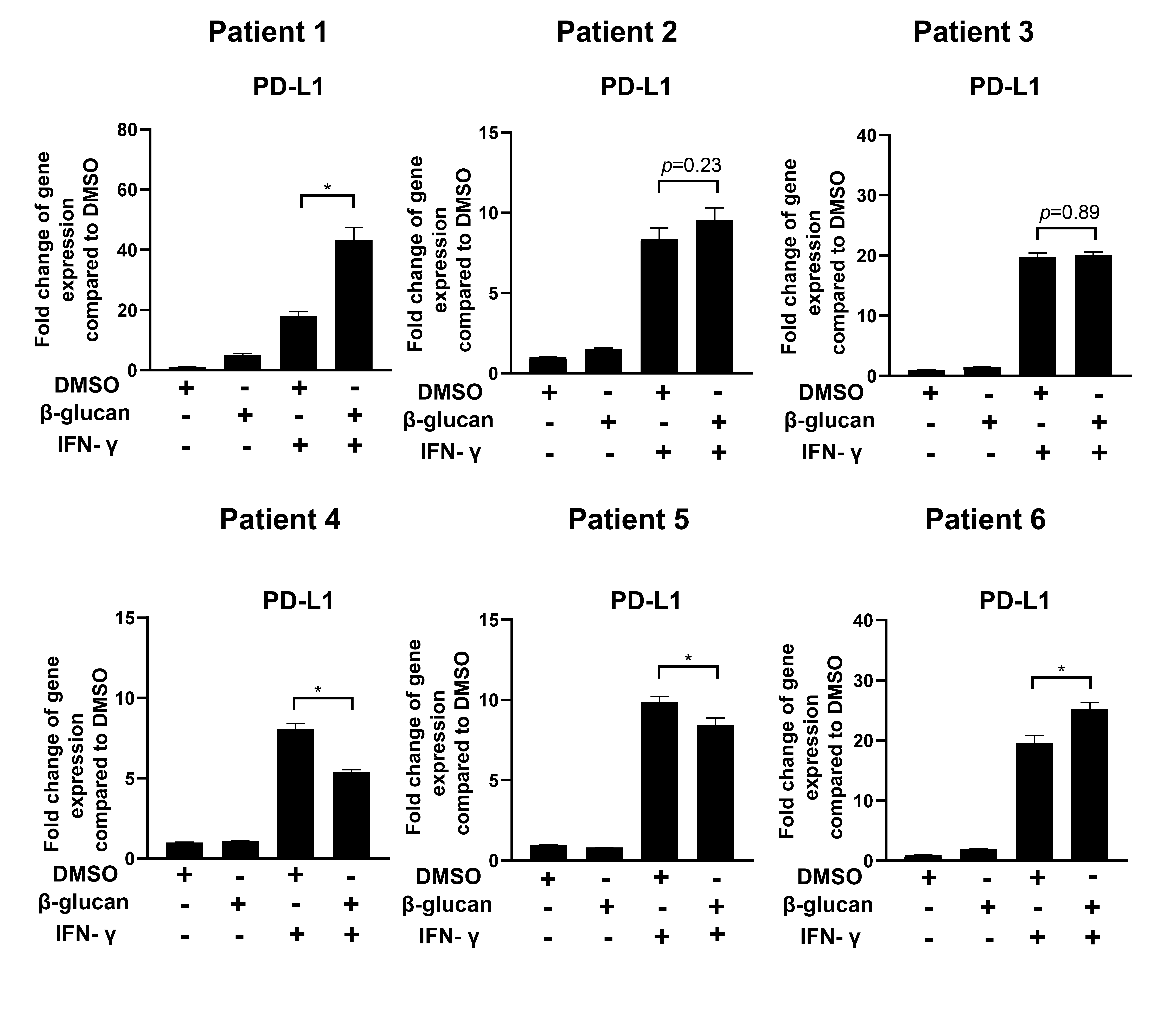

Supplement: Supplementary file 1 — Supplementary file1 (PDF 12611 kb) [file 262_2020_2781_MOESM1_ESM.docx]
